# Optical Fiber Probe Microcantilever Sensor Based on Fabry–Perot Interferometer

**DOI:** 10.3390/s22155748

**Published:** 2022-08-01

**Authors:** Yongzhang Chen, Yiwen Zheng, Haibing Xiao, Dezhi Liang, Yufeng Zhang, Yongqin Yu, Chenlin Du, Shuangchen Ruan

**Affiliations:** 1 College of New Materials and New Energies, Shenzhen Technology University, Shenzhen 518060, China; cyz867915931@163.com; 2 Key Laboratory of Advanced Optical Precision Manufacturing Technology of Guangdong Higher Education Institutes, Shenzhen Technology University, Shenzhen 518060, China; 2110416042@stumail.sztu.edu.cn (Y.Z.); liangdezhi@sztu.edu.cn (D.L.); zhangyufeng@sztu.edu.cn (Y.Z.); ruanshuangchen@sztu.edu.cn (S.R.); 3 School of Intelligent Manufacturing and Equipment, Shenzhen Institute of Information Technology, Shenzhen 518172, China; xiaohb@sziit.edu.cn

**Keywords:** fiber probe sensor, microcantilever, micromachining, Fabry–Perot cavity

## Abstract

Optical fiber Fabry–Perot sensors have long been the focus of researchers in sensing applications because of their unique advantages, including highly effective, simple light path, low cost, compact size, and easy fabrication. Microcantilever-based devices have been extensively explored in chemical and biological fields while the interrogation methods are still a challenge. The optical fiber probe microcantilever sensor is constructed with a microcantilever beam on an optical fiber, which opens the door for highly sensitive, as well as convenient readout. In this review, we summarize a wide variety of optical fiber probe microcantilever sensors based on Fabry–Perot interferometer. The operation principle of the optical fiber probe microcantilever sensor is introduced. The fabrication methods, materials, and sensing applications of an optical fiber probe microcantilever sensor with different structures are discussed in detail. The performances of different kinds of fiber probe microcantilever sensors are compared. We also prospect the possible development direction of optical fiber microcantilever sensors.

## 1. Introduction

Optical fiber sensing technology is a kind of advanced sensing technology that takes a light wave as an information carrier and optical fiber as a transmission channel to sense the measured signal from the physical, chemical, or biological [1,2,3,4]. Optical fiber sensing technology is a rapidly developing interdisciplinary field [5]. Over the past four decades, with the great need of developing the optoelectronic industry and optical fiber technology, optical fiber sensing technology has acquired enormous development. Nowadays, optical fiber sensing technology is regarded as the key development direction of the new generation of the electronic information field. Compared with conventional sensors, optical fiber sensors have attracted substantial research interest because of their incomparable inherent properties of light weight, compact size, freedom from the interference of electromagnetic irradiation, corrosion resistance, wide bandwidth, structural diversity, distributing and multiplexing capabilities, and remote operation [6,7,8,9]. These properties of optical fiber sensors make them widely used in various application fields, especially in harsh environments where electrical sensors have proven to be faulty. To date, there are various commercial applications relating to this versatile sensing technology in the fields of structural safety monitoring, aeronautics, biomedicine, petroleum, power systems, and marine monitoring [10,11,12,13,14,15].

Many different approaches utilizing optical fiber technology for sensing have been proposed. According to the sensing mechanism, optical fiber sensing technology can be roughly divided into three categories, which are intensity-modulated fiber optic sensors, wavelength-modulated fiber optic sensors, and phase-modulated fiber optic sensors. The intensity-modulated fiber optic sensor has the characteristics of a simple principle, flexible design, and low cost. It is often used to measure displacement, pressure, temperature, and vibration [16,17,18,19]. However, the fluctuation of light intensity from a light source, optical fiber, or optical device will affect the measurement accuracy to a certain extent. One way to improve the anti-interference capability of the intensity-modulated sensing system is to use multiplexing technology [20]. Wavelength-modulated fiber optic sensors mainly include fiber Bragg gratings (FBG) sensors, long period fiber gratings (LPBG) sensors, surface plasmon resonance (SPR) sensors, and lossy mode resonance (LMR) sensors [21,22,23,24]. In wavelength-modulated fiber optic sensors, sensing can be achieved by measuring the wavelength changed by the medium to be measured. They have the features of being immune to the fluctuation of light intensity, good repeatability, excellent multiplexing capability, and a mature production process. Their limitation is that the sensing area is large, which impedes its application in a space-limited environment. Phase-modulated fiber optic sensors mainly include the Michelson interferometer (MI), Mach-Zehnder interferometer (MZI), Sagnac interferometer (SI), and Fabry–Perot interferometer (FPI) [25,26,27,28]. The main characteristics of phase-modulated fiber optic sensors are compact size, high sensitivity, geometric versatility, and a wide range of application. Among the manifold optical fiber sensors, the Fabry–Perot interferometer, commonly formed by two parallel reflective mirrors separated by a certain distance, is one of the most widely deployed devices since it offers the potential for high sensitivity, high resolution, and fast response speed [29,30,31,32,33,34]. The fiber optic microcantilever sensor introduced in this paper is based on Fabry–Perot interference.

A microcantilever, commonly with a suspended beam structure anchored at one end, is the simplest micromechanical sensing component able to detect a small displacement or a tiny force [35,36]. Microcantilever sensors based on micro-electro-mechanical system (MEMS) technology have been demonstrated in the physical, chemical, medical, environmental, and material fields because of their unique advantages of simple structure, label-free detection, and high sensitivity. Depending on the state of the microcantilever, there are two operation modes that can be used for sensing target analysis: static mode and dynamic mode. Under the static mode, different compressive or tensile stresses that arise from mechanical forces or chemical reactions on the microcantilever surface can be measured by detecting the microcantilever bending. For example, a microcantilever surface modified by a thin film of metal–organic frameworks (MOFs) has been demonstrated to respond to water, methanol, and ethanol vapors [37]. In addition, Pooser et al. [38] presented an ultrasensitive displacement sensor by directly measuring the deflection of the microcantilever beam. Under the dynamic mode, the resonance frequency and mechanical quality factor are detected to accurately determine any mass change of the microcantilever. Cakmak et al. [39] fabricated two nickel microcantilevers with different geometries. The precision liquid viscosity and density are obtained via tracking the resonance frequencies of the two microcantilever with a phase-locked loop. The most widely used interrogation method for MEMS-based microcantilever sensors is the optical method including optical beam deflection and optical interferometry. In the optical beam deflection method, the nanoscale deflection could be determined by using a position sensitive optical detector to monitor the laser beam reflected from the microcantilever. This method has a high sensitivity and low noise level. However, the precision requirements for the experimental condition and critical optical alignment of the system are usually involved, which limits the utilization of microcantilever sensors in confined volume and remote sensing. An alternative solution to interrogate the signal of MEMS-based microcantilever sensors is the electrical method. For example, zinc oxide nanorods are integrated in a piezoresistive silicon microcantilever for humidity measurement and the resonant frequency shift induced by the change of relative humidity can be interrogated directly through Wheatstone bridge [40]. Although the readout system can be implanted onto the microcantilever, the sensitivity of the electrical method is less accurate than the optical method. Moreover, there is still a challenge for MEMS-based microcantilever sensors using electrical interrogation operating in conductive liquids, electromagnetic noisy environments, explosive gases, or extreme temperatures. To solve the above problems, Ianuzzui [41] proposed a novel design in 2006 that fabricated the microcantilever on the optical fiber-top, forming an extrinsic Fabry–Perot sensor. This unique design provides the advantages from both microcantilever and optical fiber sensing. From then on, a variety of optical fiber microcantilever sensors, different fabrication methods, and application scenarios have been developed. 

To our knowledge, there are few reviews focused on optical fiber microcantilever sensors. In this paper, a brief review of optical fiber microcantilever sensor based on Fabry–Perot interference is presented. The aim of this review is to outline the basics of the optical fiber microcantilever sensor and draw attention to the potential of utilizing optical fiber microcantilever-based devices for sensing applications. The background of optical fiber sensors and the characteristics of microcantilevers are introduced briefly in the introduction section. In the next section, the operation principle and readout method are illustrated. In the third section, the fiber-top microcantilever sensor will be described in detail, with respect to its different structures, a variety of fabrication methods, and various applications. The ferrule-top microcantilever sensor and the other microcantilever sensors will be summarized in the fourth and fifth sections, respectively. Different kinds of fiber probe microcantilever sensors are compared in the sixth section. Finally, a conclusion about the optical fiber microcantilever sensor and the outlook for the development potential in sensing applications are provided.

## 2. Fundamental of Optical Fiber Microcantilever Sensor 

**Figure 1:**
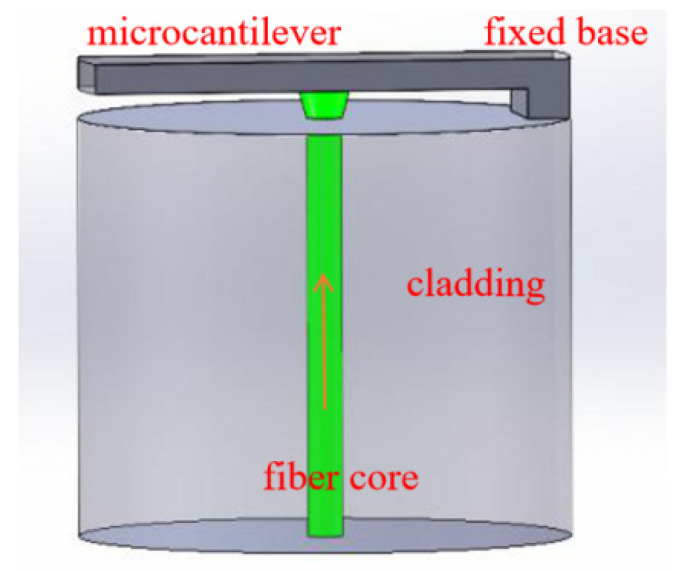
The configuration of an optical fiber microcantilever sensor.

The schematic diagram of the optical fiber microcantilever is illustrated in Figure 1. It comprises a rectangular mechanical beam and a fixed base at the cleaved end edge of an optical fiber. The microcantilever beam is suspended directly above the optical fiber core. Light transmitted from the other end of the optical fiber partially reflects when it reaches the end of the fiber and the remainder passes though the fiber end, illuminating the cantilever beam. Because of the refractive index mismatch, the light reflected from cantilever beam couples back to the optical fiber and interferes with the reflected light at the fiber end. Therefore, FP interference is formed between the cantilever beam and the fiber end face. The sensing principle of this type microcantilever depends on the deflection of the free end of the microcantilever. Compared to the FPI sensor based on an enclosed diaphragm, an optical fiber microcantilever sensor has a large deformation under the same conditions, which leads to a higher sensitivity and a large dynamic response range. When external physical, chemical, or biological factors cause deflection of the microcantilever beam, the cavity length of the FPI will change. Consequently, the external signal can be accurately measured by demodulating the deflection of the cantilever beam. According to the Stoney equation [42], the rectangular microcantilever deflection is proportional to the differential surface stress on microcantilever [43], which is given by:

(1) $$z=\frac{3L^{2}\left(1-\mu \right)}{Et^{2}}\Delta \sigma$$

where z is the deflection, L is the length of the rectangular microcantilever, *μ* and E are the Poisson’s ratio and Young’s modulus for the material of microcantilever, respectively, t represents the microcantilever thickness, and ∆σ represents the differential surface stress on the microcantilever.

**Figure 2:**
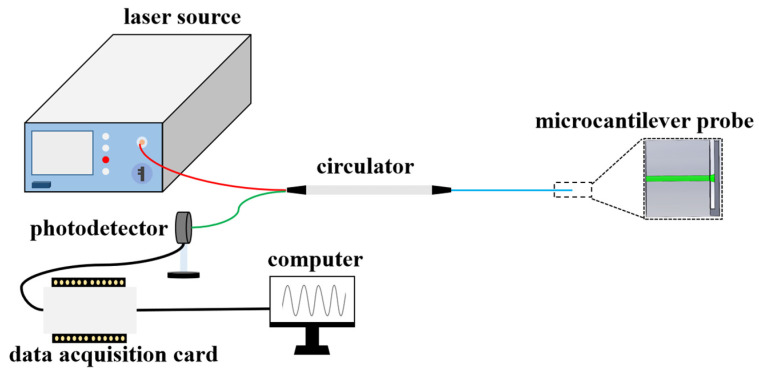
Experimental setup of the microcantilever probe readout system.

To detect the optical interference signals from the microcantilever probe, a common readout system is shown in Figure 2. The light from the laser source (usually broadband laser or tunable laser) is coupled to the microcantilever probe through a circulator and generates FP interference as described above. The reflected interference signals can be measured by a photodetector and then converted into a digital signal through a data acquisition card. Finally, the signal can be demodulated by computer program. Taking advantage of the ultra-compact size of microcantilever and the flexibility of the optical readout system, one can remotely detect any factors acting on the microcantilever in a small volume. The reflected optical intensity I can be expressed as:

(2) $$I=I_{0}\left[1+Vcos\left(\frac{4\pi nd}{\lambda }+\Phi_{0}\right)\right]$$

where I_0_ is the input optical intensity, Φ_0_ is a constant phase shift, λ is the wavelength of the laser, V represents the fringe visibility, n is the refractive index, and d represents the distance between fiber end face and microcantilever beam.

## 3. Fiber-Top Microcantilever Sensor

### 3.1. Focused Ion Beam Milling

**Figure 3:**
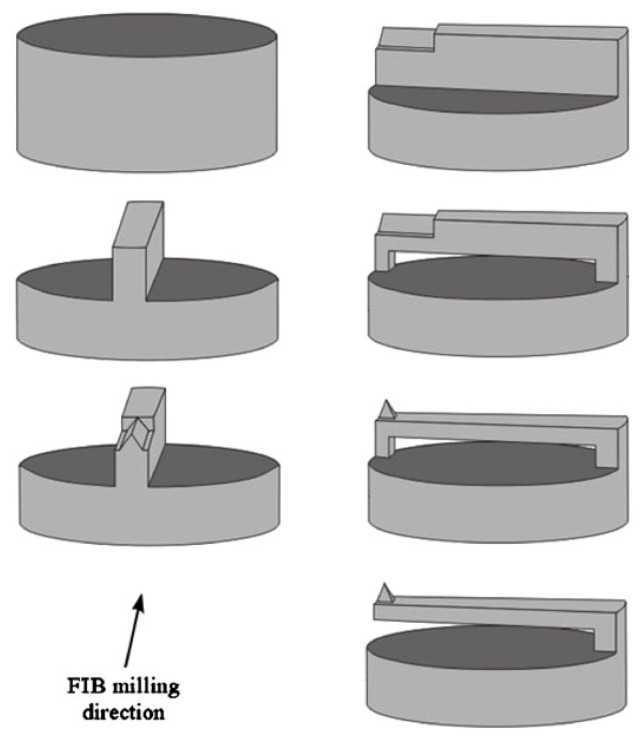
The fabrication process of a fiber-top microcantilever using FIB milling [44].

The fiber-top microcantilever can be machined by focused ion beam (FIB) milling, chemical etching, laser ablation, photolithography, or two-photon polymerization. In 2006, Ianuzzui et al. [41] pioneered the fabrication of a microcantilever at the optical fiber-top using FIB technology. The fabrication process includes stripping the fiber jacket, cleaving the fiber, coating the metallic layer, and FIB carving. Combining the features of a microcantilever and fiber optic sensor, the fiber-top microcantilever is a kind of monolithic, plug-and-play, and self-alignment device that has the potential for a wide range of applications, including atomic force microscopy (AFM) and temperature measurement. Moreover, due to the all-silicon structure, the fiber-top microcantilever is especially suitable for sensing measurements in extreme environments. For the implementation of fiber-top microcantilevers in scanning probe microscopy, the same group optimized the microcantilever beam structure with a sharp pyramidal tip equipped at the top of the microcantilever end [44,45]. The fabrication process of this fiber-top microcantilever using FIB milling technology is presented in Figure 3. First, a chromium film of 5 nm and a palladium film of 20 nm were sputtered onto the fiber top for the prevention of electrostatic charge accumulation during the FIB milling. Then, both sides of the metal-coated optical fiber was milled to form a ridged part in the middle of the fiber end face. A triangular edge was carved above the ridged part. Next, the fiber was rotated 90° to pierce and remove the topside of the ridge. The final pyramidal tip was fine machined at a low current intensity. The results found that the performances of this fiber-top probe employed as a contact-mode AFM was comparable to that of commercial AFM and, in the meantime, offered the advantages of being simple and easy to use. Similar to this work, Tiribilli et al. [46] proposed a hybrid fiber-top microcantilever. They fabricated the sharp pyramidal tip directly above the optical fiber core and removed a large part of the fiber from the edge opposite to the anchor point of the cantilever. The experimental results showed that the hybrid probe can both serve as a scanning near-field optical microscope (SNOM) and AFM. The SNOM signal from the evanescent optical field of an illuminated prism was detected by a photomultiplier through the central pyramidal tip, whereas the AFM information was obtained by demodulating the bending of the microcantilever. 

The fiber-top microcantilevers mentioned above are based on a single-mode fiber (SMF) with a diameter of 125 μm. FIB technology has the capability to micromachine a fiber sensor in a smaller volume with superior surface quality, which opened a window of opportunity for measurements at micron-scaled dimensions [47,48]. In 2016, fiber-top microcantilevers with 20 μm diameters and 50 μm diameters were fabricated on etched fiber using FIB milling technology by Moxi [49]. They investigated the performance of the fiber-top probe in AFM imaging mode both in air and water, and employed the Oliver and Pharr method to calculate the Young modulus of the sample in indentation mode. However, the ultracompact fiber-top microcantilever has some drawbacks, such as image distortions, being too fragile in contact with the sample, and high production cost, which restricted its further application. 

In addition to measuring physical quantities, fiber-top microcantilevers can also be applied for the detection of chemical species. Using a similar FIB fabrication method and thermal evaporation, a fiber-top microcantilever sensor with a thin palladium layer for hydrogen detection was obtained [50]. When the microcantilever is exposed to hydrogen, hydrogenation of the palladium layer gives rise to a mechanical strain that causes deflection of the microcantilever. Subnanometer deflection was successfully detected by means of optical interferometry. The great improvement of this fiber-top microcantilever chemical sensor with respect to other chemical sensors is that it can work in extreme environments, taking advantage of an all-silica structure, and it does not require any optical alignment because of the monolithic structure.

### 3.2. Chemical Etching

**Figure 4:**
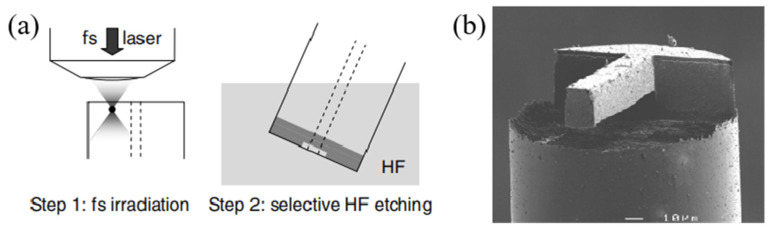
(**a**) Fabrication process of femtosecond-laser-assisted chemical etching. (**b**) SEM image of the fiber-top microcantilever [54].

Although FIB milling technology has proved to be suitable for micromachining, with the main advantage of high machining accuracy, it is high cost and time consuming to FIB micromachine fiber-top microcantilevers because of the low material removal rate of the FIB process. To mitigate this problem, an alternative micromachining technique is femtosecond laser (fs-laser)-assisted chemical etching, which has been successfully corroborated to be an effective two-step process for creating optofluidic microchips and free-space optical components [51,52,53]. The basic principle of this micromachining technique is based on the fused silica having a much higher etching rate of chemical solutions after fs-laser irradiation. In 2007, Said et al. [54] used fs-laser-assisted chemical etching to product a microcantilever at the end of an optical fiber. In this paper, a Ti:sapphire fs-laser produced a focused pulse sufficient to alter the chemistry selectivity of the glass but not enough to ablate it. After being irradiated by the fs-laser pulses, the fiber was immersed in an aqueous solution of hydrofluoric acid to selectively remove exposure areas. The fabrication process and scanning electron microscope (SEM) image of the fiber-top microcantilever device manufactured by fs-laser-assisted chemical etching is shown in Figure 4. The results indicated that the two-step process has lower fabrication cost and higher material removal rate in comparison with FIB milling, which may make it more adaptable for quantity production. However, the thickness of the microcantilever is larger than those machined by FIB milling and the surface of the machined region is too rough, so most of the light containing the sensing information is scattered. The machining time of fs-laser-assisted chemical etching has been reduced with respect to FIB milling, but it still took 90 min to complete the entire device.

### 3.3. Picosecond-Laser Ablation

**Figure 5:**
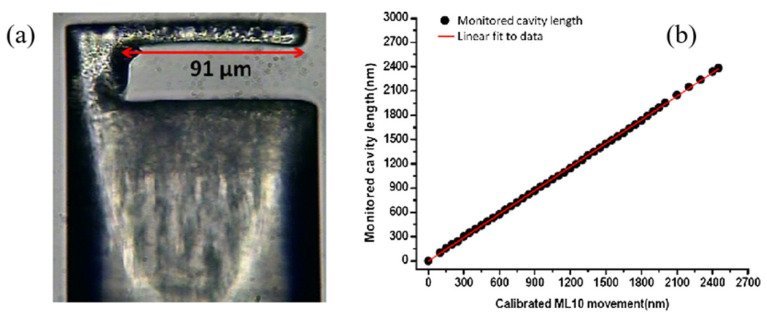
(**a**) Laser-machined fiber microcantilever. (**b**) Displacement performance [55].

Another alternative micromachining solution to fabricate a fiber-top microcantilever is picosecond-laser (ps-laser) ablation. In 2013, Frank et al. [55] demonstrated the approach to manufacturing microcantilever sensing components on the top of optical fibers by the employment of a commercial ps-laser system. By optimizing laser parameters, compensating taper angle, minimizing re-deposited debris, and introducing a polishing process, the microcantilever surface parallel to the end surface of the fiber with optical quality was obtained. The experiment results showed that the microcantilever-adopted picosecond-laser manufacturing technology can act as a displacement sensor with a range of more than 3 μm, as can be seen in Figure 5. The ps-laser direct machining greatly reduces the processing time and has a good processing accuracy with respect to chemical etching methods. Exploiting the high machining speed of ps-laser direct machining and the excellent surface roughness of FIB milling, Li et al. [56] proposed a microcantilever temperature sensor with a high sensitivity of 40.2 nm/°C in the range from room temperature to 500 °C. Similar to [55], a fiber-top microcantilever that was 110 μm long, 18 μm wide and 8 μm thick was first machined by ps-laser machining. Next, FIB milling was introduced to polish the microcantilever surface, reducing the thickness and improving the mechanical sensitivity. After polishing, the surface roughness can reach nm range. For sensing temperature, aluminum was coated on the top side of the microcantilever because of its high thermal expansion coefficient and a phase recovery algorithm was applied to measure the cavity length with a resolution within 2–3 nm. The combination of ps-laser ablation and FIB milling paves the way for rapid production of fiber-top microcantilevers with small surface roughness. In 2015, the same team expanded the application of fiber-top microcantilevers from temperature to pH sensing [43]. The fiber-top microcantilever pH sensor processed by ps-laser ablation and FIB milling has dimensions of 112 μm, 15 μm, and 1.5 μm, which is capable of pH sensing in a space-limited environment. For sensing pH, the microcantilever was modified by coating different functional layers on different sides of the microcantilever. In this case, the change of pH caused a different surface strain on each side of the microcantilever, which resulted in the defection of the microcantilever. The results confirmed that the Al_2_O_3_/Au-functionalized cantilever is sensitive from pH 7.0 to pH 9.0 with ~100 nm/pH sensitivity and the MHA-functionalized cantilever is sensitive from pH 4.0 to pH 10.0 with ~15 nm/pH sensitivity.

### 3.4. Photolithography

Up to now, the above fabrication technologies, namely FIB milling, chemical etching, and laser ablation, are based on removing the material of optical fibers to form a microcantilever structure as sensing components. Photolithography, as a conventional top-down micromachining, is the most important process in semiconductor manufacturing and also works for the fabrication of fiber-top microcantilevers. For example, Gavan et al. [57] took advantage of photolithography to fabricate a low-mass gold fiber-top microcantilever. The procedure of photolithography can be described simply as coating photoresist, UV light exposing resist development, deposition, and wet etching. Due to the high processing accuracy of photolithography and the manufacturing operation based on depositing instead of removing substances, the thickness of the metallic microcantilever is only 350 nm, which makes it eminently suitable for mass sensing. The result showed that the low-mass gold fiber-top microcantilever has a mass sensitivity of 5 ag/Hz with a mechanical quality factor of 98. On the basis of [57], Rector et al. [58] optimized the fabrication procedure of photolithography to improve the yield and increase the reliability.

### 3.5. Two-Photon Polymerization

**Figure 6:**
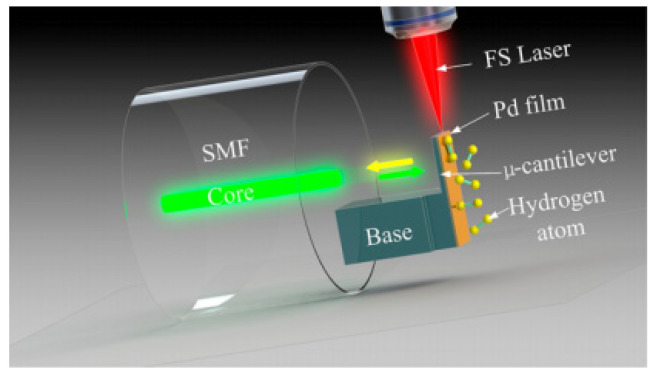
The polymer-based fiber-top microcantilever fabricated by TPP technology [59].

A new additive manufacturing method for a fiber-top polymer-based microcantilever hydrogen sensor was proposed by Xiong et al. [59], as shown in Figure 6. This is the first time that femtosecond laser-induced two-photon polymerization (TPP) has been used to develop a polymer-based microcantilever in medical and environmental applications. There are many individual advantages of TPP technology, including its ability to be flexibly manufactured and its high machining accuracy, which make it widely used in micromachines, photonics, biomedicine, and microfluidics [60,61,62,63]. The fabrication process of the polymer-based microcantilever using TPP can be summarized in four steps. First, the cleaved fiber tip is immersed in the negative photoresist. Second, a low-power femtosecond laser with wavelength of 1026 nm is focused on the negative photoresist and scans to polymerize a microcantilever structure on the fiber top. After that, the remaining photoresist is washed away by a mixture solution of acetone and isopropyl alcohol. Last, the upper surface of polymer-based microcantilever beam is coated with palladium by a magnetron sputtering device. Experimental results reveal that such a hydrogen sensor has a nonlinear response based on wavelength shift and a high repeatability with hydrogen concentrations ranging from 0 to 4.5%. Moreover, the short response time of the fiber-top microcantilever, which is about 13.5 s at 4% hydrogen concentration, can satisfy the requirements of medical and biological applications. However, the polymer-based fiber-top microcantilevers coated with palladium have some limitations, such as the temperature cross-sensitivity caused by the thermal expansion effect, crosstalk from other active gases in the mixture, and a monotonous structure. In 2022, the same research group extended the structures of the polymer microcantilever [64]. Three different forms, namely rectangular solid, rectangular hollow, and triangular microcantilever, were printed directly on an optical fiber end face by means of TTP additive manufacturing. They found that the rectangular hollow shape cantilever coated with 60 nm thick palladium has the maximum sensitivity and the response time was improved to 5.3 s at 4% hydrogen concentration.

## 4. Ferrule-Top Microcantilever Sensor

Ferrule-top microcantilevers are another kind of microcantilever fiber sensors that can be obtained by carving a microcantilever on the top of a glass ferrule or mounting a microcantilever film above the ceramic ferrule. The sensing mechanism is similar to that of a fiber-top microcantilever, which is also based on FPI. The FP cavity is formed by the cleaved end of the fiber and the microcantilever. A ferrule-top sensor has the main advantages of a fiber-top cantilever including being monolithic, having no alignment, and being versatile. However, by plugging the glass/ceramic ferrule at the end of a cleaved fiber, the size of the whole sensor head increases by an order of magnitude. One can produce a microcantilever beam on the upper side of the ferrule with far more efficiency, low production cost, and more adjusting to series production, such as ps-laser ablation or wire saw. According to the materials of ferrule, the ferrule-top microcantilever sensors are classified as glass ferrule-top microcantilever sensors and ceramic ferrule-top microcantilever sensors.

### 4.1. Glass Ferrule-Top Microcantilever Sensor

**Figure 7:**
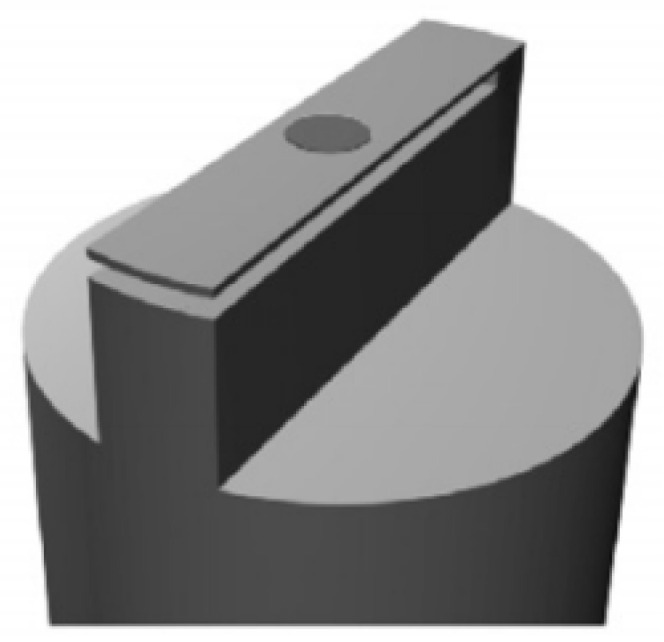
3D model of the glass ferrule-top cantilever [65].

In 2010, Gruca et al. [65] first presented the concept of a ferrule-top microcantilever fiber sensor. The ferrule-top microcantilever fiber sensor consists of a glass ferrule and a readout fiber as illustrated in Figure 7. At the top of the ferrule, a microcantilever beam 1.6 mm long, 200 μm wide, and 30 μm thick is laser-machined. The complete fabrication procedure takes about 60 min, which proves that the ps-laser ablation is efficient for fabricating ferrule-top cantilevers. The SMF is slid into the center hole of the ferrule and glued. The results indicated that the resonant frequency and the quality factor of the device are 11.7 kHz and 225, respectively, for the first resonant mode in air.

Cipullo et al. [66] applied the glass ferrule-top microcantilever fiber sensor to work in static mode to measure velocity in air flows. Experimental results showed that the sensitivity of the prototype system is 82 mV/(m/s), with the minimum detectable flow velocity change in the order of 0.1 m/s. Moreover, the glass ferrule-top sensor shows no hysteresis and a good short-term repeatability, but it is still affected by long-term drifts. In 2012, the same group used the finite element method (FEM) to numerically study the airflow speed sensor based on a ferrule-top microcantilever and compare with experimental results [67]. In this numerical model, the air inflow is considered laminar. Air flow through the glass microcantilever causes bending of the microcantilever and therefore changes the cavity length. Numerical results reveal that part of the airflow enters the gap between microcantilever and ferrule. Therefore, a positive pressure exists on the back face of the microcantilever, limiting the sensitivity of the ferrule-top airflow speed sensor, which is aggressive with the experimental results. 

**Figure 8:**
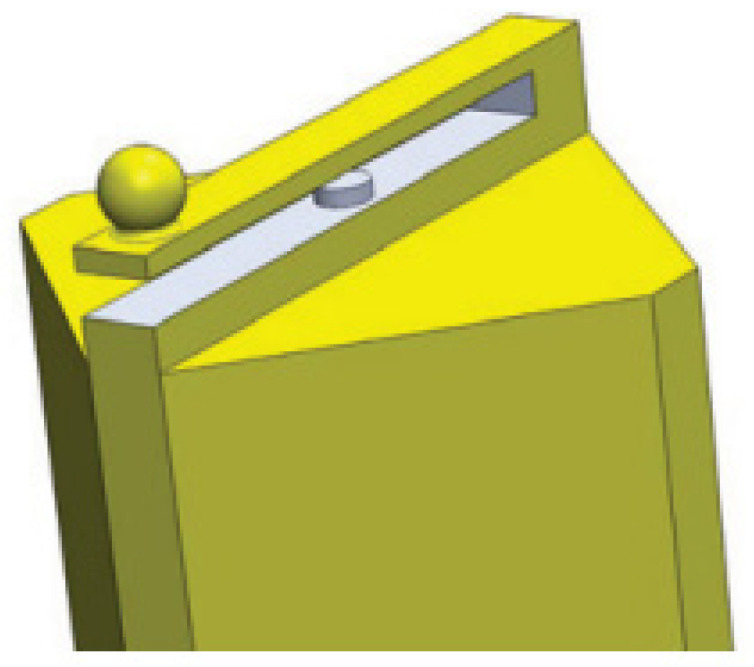
Ferrule-top cantilever for Casimir force measurements [68].

Zuurbier et al. [68] fabricated a diagonal cantilever on the top of a rectangular glass ferrule using laser ablation and proposed a fiber sensor for the measurement of Casimir force. The geometry of this device is shown in Figure 8. A gold-coated sphere with a diameter of 200 μm is attached to the hanging end of the microcantilever beam using a small drop of epoxy. The ferrule-top microcantilever is 3.4 mm long, 200 μm wide, and 40 μm thick in size and the gap between the microcantilever and the fiber end is about 100 μm. When the gold-coated sphere slowly approaches a gold-coated plate by means of the piezoelectric stage, the result shows that the Casimir force gradient is a function of the separation in the scope of 160 nm to 200 nm with the standard deviation of 2.5 N/m^2^. It means that the ferrule-top cantilever has the capability to accurately measure Casimir force. 

Chavan et al. [69] developed a compact AFM setup based on ferrule-top fiber sensor. The AFM that works in contact mode can image in air and in liquids with high sensitivity. A v-groove on the side of the borosilicate glass ferrule is machined by laser ablation. A conical tip that is 8 μm high with a 100 nm radius is glued to the free-hanging end of the microcantilever for contacting with grating samples. The glass ferrule-top microcantilever with spring constants of 8 N/m and resonance frequencies of 5 kHz is coated with a 30 nm thick Au layer to allow more reflected light to couple back to the readout fiber. The experimental results show that the ferrule-top AFM can correctly reproduce the shape of the calibration grating with a certain quality of the image in air and water. Furthermore, the same author demonstrated that a ferrule-top microcantilever probe can also provide tapping mode images at cryogenic temperatures [70].

In 2012, Schenato et al. [71] used a glass ferrule-top microcantilever fiber sensor to monitor the precursory acoustic emissions in unstable rock masses. The performance of ferrule-top cantilever sensor was compared with the fiber-coil sensor and piezoelectric transducer. To simulate realistic acoustic emission signals, a 5 mm diameter steel ball was dropped on the top of a block 50 × 50 × 15 cm^3^ in size. The sensors were screwed in a threaded anchor that was internally glued at the bottom of the block for acoustic coupling and impedance matching. The comparative experimental study has found that the ferrule-top fiber sensor is the most sensitive sensor, while the temporal resolution is the worst because of the damped oscillation. In addition, a ferrule-top fiber sensor is more sensitive to volume waves, which proved its feasibility in the detection of acoustic emissions in rockfall events.

Dhwajal et al. [72] improved the fabrication process of the glass ferrule-top microcantilever. They used a wire cutter to remove bulk material at the beginning of the process instead of ps-laser ablation. Laser ablation is still used for the rest of the process to obtain the microcantilever structure. This method can slightly shorten the manufacturing time, but the sample needs to transfer from the wire-cutter platform to the laser ablation system. The ferrule-top cantilever was equipped with a sharp tip to simultaneously probe the topography and collect/emit light. Such a setup combined the function of AFM and SNOM. Using a similar microcantilever probe, one can simultaneously implement optical coherence elastography depth sensing and atomic force microscope indentation [73].

In 2013, Gruca et al. [74] demonstrated that the glass ferrule-top microcantilever can be actuated with light based on a photothermal effect. The fabrication process is similar to ref. [72] using the combination of wire saw and laser ablation. The glass microcantilever will vibrate at a specific frequency when it is illuminated by an intensity-modulated laser. The proof-of-concept experiments confirm that the ferrule-top microcantilever based on the excitation scheme can be applied to the measurement of humidity and pressure via the detection of the vibration frequency of the microcantilever.

**Figure 9:**
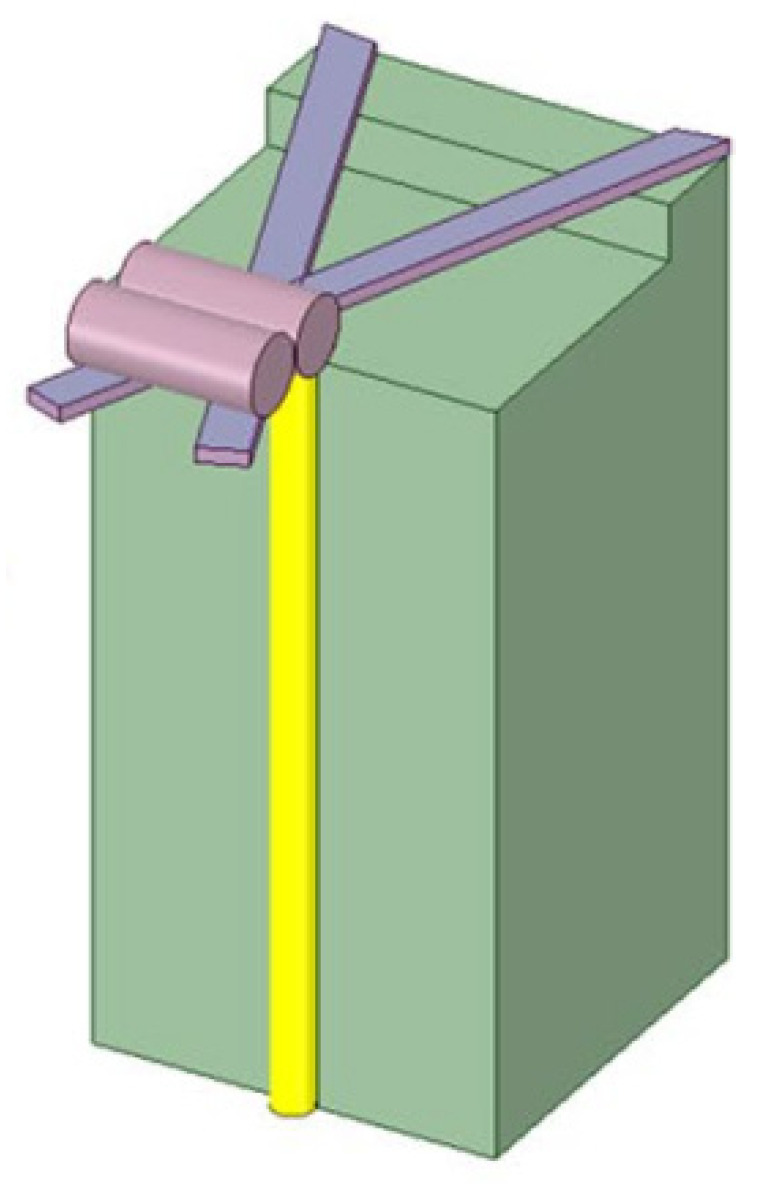
Seismic accelerometer sensor based on X-shaped cantilever [75].

In 2018, Pisco et al. [75] designed a seismic accelerometer sensor that relied on an X-shaped microcantilever, as shown in Figure 9. Instead of using ps-laser ablation to carve the glass ferrule, the seismic accelerometer was obtained by cutting out a single ridge using the wire cutter. Then two glass ribbons with 200 μm widths and 20 μm thicknesses were fixed to the ridge. The copper pieces positioned at the top of the X-shaped microcantilever functioned as proof mass to tune the sensor response. The SMF fixed in the side groove was used to measure the resonance frequency of the X-shaped microcantilever. This seismic accelerometer has successfully sensed and recorded the ground acceleration associated with real earthquakes, which verified the reliability of the X-shaped microcantilever accelerometer in seismic-wave detection. In 2020, the accelerometers based on ferrule-top microcantilevers were further improved in terms of numerical simulations and experimental realization by Francesco [76,]. Two accelerometers with different geometrical features have been fabricated. Each of them exhibited a sensitivity of about 0.1 nm/(m/s^2^) at 5 kHz.

### 4.2. Ceramic Ferrule-Top Microcantilever Sensor

A ceramic ferrule-top microcantilever sensor is composed of optical fiber, ceramic ferrule, microcantilever film, and a housing shell. Acoustic detection is the main application field of ceramic ferrule-top cantilever sensors. The materials of cantilever film can be divided into polymer, stainless steel, and silicon.

Li et al. [77] introduced ferrule-top technology to fabricate microcantilever-based biosensors for food pathogen detection. A polyimide microcantilever is produced by ns-laser machining and then boned to the top of a ceramic ferrule with a donut-shape photoresist as a supporting layer. The polymer microcantilever with a length of 1.4 mm, a width of 300 μm, and a thickness of 25 μm is metallized with gold to enhance reflectivity. Subsequently, different biomaterials are attached on the gold-coated microcantilever surface via a standard self-assembled monolayer (SAM) process. These biomaterials are used to transfer the biomolecular interaction to the detectable nanomechanical force of the microcantilever, which will determine the selectivity and sensitivity of the ferrule-top optical fiber biosensor. The experimental results demonstrated that this biosensor probe has a minimum detection level of ~10 nM streptavidin. In addition, a minimum level of less than 105 cfu/mL was attained for the detection of Listeria food pathogen concentrations when the selected Listeria capture antibody was coated on the polymer cantilevers.

In 2018, Chen et al. [78] fabricated a ceramic ferrule-top microcantilever microphone using 304 stainless steel as the microcantilever material. The ferrule-top microcantilever microphone consists of a readout fiber, a ceramic ferrule, a stainless shell, and a stainless steel microcantilever that is processed by a laser marking machine. The fast demodulated white-light interferometry (WLI)-based demodulation method is applied to measure the absolute length between the fiber end and microcantilever. Experimental results demonstrated that the ferrule-top microcantilever microphone has a highly sensitivity of 211.2 nm/Pa at 1 kHz with a large dynamic range of acoustic pressure response. Moreover, the high SNR and the high stability of the microphone make it suitable for resonance or non-resonance photoacoustic spectroscopy-based trace gas detection. The high sensitivity measurement of the trace acetylene, ammonia, and methane experiment has confirmed the ability of stainless-steel cantilever film with a 10 μm thickness to be an acoustic sensing component in all-optical photoacoustic spectroscopy [79,80,81,82]. The minimum detection limit of trace acetylene, ammonia, and methane are demonstrated to be 71 ppt, 3.2 ppb, and 15.9 ppb, respectively. Furthermore, the measurement of the acoustic signal and temperature vibration can be simultaneously obtained by a single stainless-steel microcantilever-based optical fiber sensor [83]. The length of the extrinsic FP cavity, which is the distance between the end face of the optical fiber and the inner surface of the cantilever beam, changes slowly with temperature while it periodically deflects with acoustic pressure. Therefore, the acoustic signal and temperature vibration can be measured by demodulating the DC and AC components of the extrinsic FP cavity. The acoustic pressure sensitivity and the temperature sensitivity are 193.8 nm/Pa at 1kHz and 83 nm/°C, respectively. 

For in situ detection of dissolved gas in oil, a highly sensitive optical fiber photoacoustic sensor was designed and experimentally verified [84]. The separation membrane allows the dissolved gas to be separated from oil. The ceramic ferrule-top stainless-steel microcantilever microphone was used to probe the photoacoustic signal proportional to the dissolved gas concentration. The experimental results demonstrated that the detection limit of dissolved acetylene gas is 0.5 μL/L with a response time of 1.8 h at 50 °C.

**Figure 10:**
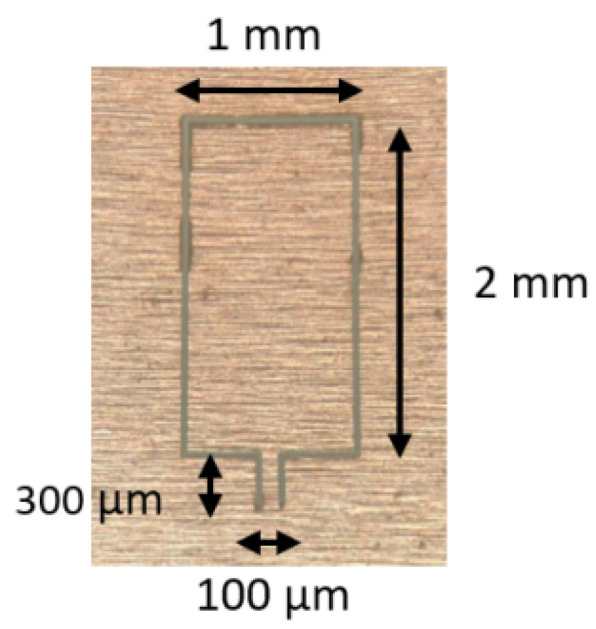
Microscope image of the hinged cantilever [86].

In 2020, a similar structure of a microcantilever-based acoustic sensor was proposed by Xin et al. [85] for the detection of CO_2_ concentrations. Attributed to the resonance enhancement of the cantilever-based microphone and second-harmonic detection, the detection limit of CO_2_ concentration reaches 0.044 ppm. Cantilever-enhanced photoacoustic spectroscopy (CEPAS) technology can effectively improve the sensitivity of trace gas photoacoustic signals. The all-optical FP acoustic sensor with a cantilever structure has the merits of simple structure, compact size, and high stability of optical path compared to traditional mechanical cantilever photoacoustic spectroscopy systems. Thomas et al. [86] designed and fabricated a hinged microcantilever-based microphone for trace NO detection in a nitrogen atmosphere. The stainless-steel hinged microcantilever is manufactured by laser cutting, as shown in Figure 10. Then, the hinged microcantilever is assembled on a metallic support to form an FP acoustic transducer with an optical fiber. Such a design ensures a larger displacement of the free-hanging end of the cantilever, which means the higher sensitivity of an acoustic sensor. Experimental results show that the hinged microcantilever-based microphone has a high acoustic sensitivity of 630 mV/Pa and the detection limit of NO is as low as 15 ppb. 

In addition to stainless steel microcantilevers, silicon can also be used for ceramic ferrule-top microcantilevers. In 2021, Gong et al. [87] proposed a silicon microcantilever-based ferrule-top acoustic sensor. A 3 μm thick silicon microcantilever was fabricated on the 400 μm thick silicon-on-insulator (SOI) wafer through MEMS technology. The FP cavity length of the ferrule-top acoustic sensor is estimated to be 330.76 μm using an ultra-high-speed spectrum demodulation method. Experimental results indicated that this sensor has a relatively flat frequency response in a wide range from 20 Hz to 13 kHz. Moreover, the signal-to-noise ratio SNR and sensitivity of this silicon microcantilever-based ferrule-top acoustic sensor reach 71.81 dB and 950 nm/Pa, respectively. Another silicon microcantilever with a larger geometry was fabricated by Guo et al. [88]. Additionally, a layer of gold film was coated on the silicon microcantilever to improve the contrast of the reflection spectrum. Compared to ref. [87], the larger geometry made it more sensitive to acoustic pressure, with the ultrahigh acoustic pressure sensitivity of 1753 nm/Pa at the frequency of 1 kHz. The minimum detectable pressure (MDP) level of this acoustic sensor based on a gold-plated silicon cantilever is 0.21 μPa/Hz^1/2^. The experiment found the MEMS technology can be adopted to batch-produce silicon microcantilevers with lower cost. In 2022, Guo et al. [89] successfully demonstrated the photoacoustic signal detection using a silicon microcantilever-based acoustic fiber sensor. The photoacoustic signal was generated by periodic light absorption of C_2_H_2_ in the non-resonance photoacoustic cell. The acoustic performance test found that the minimum detection limit of C_2_H_2_ is 199.8 ppt with an average time of 60 s.

## 5. Other Microcantilever Sensors

**Figure 11:**
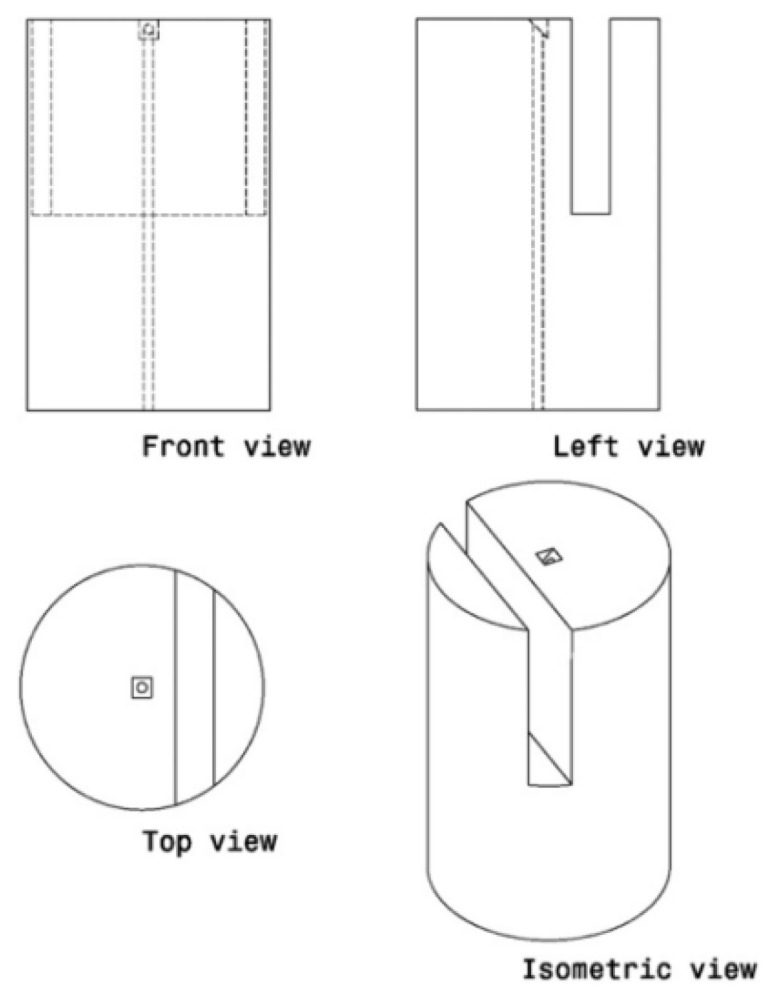
Three-view drawings of the fiber-side interferometer sensor [90].

Except for the literature mentioned above, Sun et al. [90] proposed a prototype of a fiber-side microcantilever fabricated by a three-dimensional deterministic FIB machining technique. As shown in Figure 11, a deep groove was machined in the top of a single-mode fiber, which formed a Fabry–Perot cavity. The 45° reflective mirror (10 μm × 10 μm) at the end of the fiber core was carved and polished with a surface roughness of about 10 nm. The interferometry experiment found that the measurement resolution of the Fabry–Perot cavity is around 50 nm through fast Fourier transform analysis. Another optical fiber side-cantilever was demonstrated by Li [91] for acceleration measurement. Different from ref. [90], Li employed a ps-laser to produce the deep groove with high-speed material removal, while the microcantilever surface and the 45° reflective mirror was still machined by FIB milling to obtain a smooth optical surface. The performance test indicated that the side-microcantilever accelerator has an acceleration measurement range from 0 g to 6 g with a resolution of 0.01 g.

In 2016, Liu et al. [92] presented an all-silica cantilever-based optical fiber acoustic sensor. The cleaved end of the fiber and the silica cantilever at the end of a capillary tube form a Fabry–Perot cavity. The interference signal from the FP cavity will be modulated by an acoustic wave and demodulated by interferometry. By optimizing the femtosecond laser processing parameters, a rectangular-shaped silica cantilever with a thickness of 5 μm was achieved. The sensor has a frequency response range from 100 Hz to 3.2 Hz. Moreover, the all-silica cantilever-based acoustic sensor with an open cavity can be used as a hydrophone [93].

**Figure 12:**
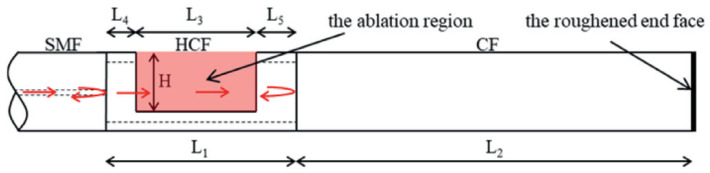
Fiber-optic microcantilever-based vibration sensor [94].

**Figure 13:**
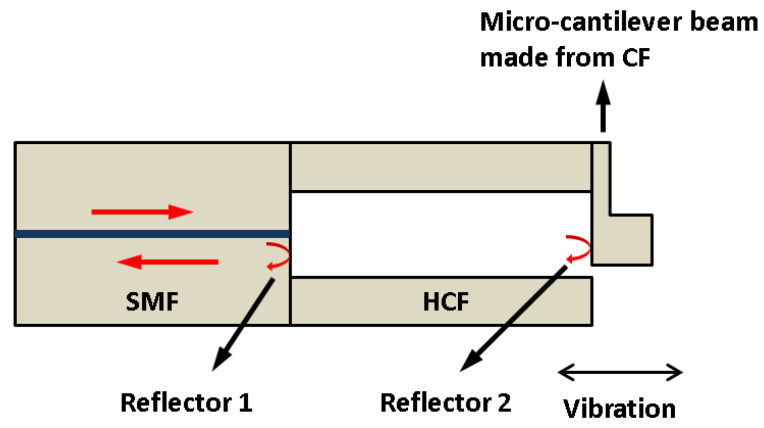
The configuration of on-fiber EFPI vibration sensor [96].

In 2018, Zhang et al. [94] demonstrated a fiber-optic microcantilever-based vibration sensor. The sensor was formed by a section of hollow-core fiber (HCF) sandwiched by a SMF and a coreless fiber (CF). The microcantilever was obtained by milling parts of HCF using fs-laser, as shown in Figure 12, which can be used to detect a vibration parallel to the direction of the optical fiber axial. The results indicated that the sensor has a high sensitivity of 20.678 mV/g at 500 Hz in the acceleration range of 0–10 g. A fiber-optic accelerometer that can be used to detect a vibration perpendicular to the direction of the optical fiber axial was also proposed [95]. The cantilever beam glued with a silica inertial mass was fabricated by fs-laser. Experimental results found that the inertial mass greatly enhanced the sensitivity of the accelerometer with a high sensitivity of 2.9 nm/g at 500 Hz and a response range from 0 g to 3 g. However, the large dimensions and the usage of polymeric adhesives may limit the response range and long-term stability of the fiber-optic accelerometer. Based on an all-fiber design, Weiyi et al. [96] presented a miniature microcantilever vibration sensor with a sensor diameter of 125 μm, as shown in Figure 13. The cantilever was obtained by an fs-laser processing the coreless fiber that was spliced on the hollow-core fiber. Experimental results demonstrated that the all-fiber microcantilever sensor has an acceleration sensitivity of 1.1 mV/g at 300 Hz in a wide response range of 0.5–5 g.

## 6. Comparison of Optical Fiber Probe Microcantilever Sensor

The performance comparison of the optical fiber probe microcantilever sensor in terms of structural type, fabrication method, microcantilever size, and sensing application is listed in Table 1. After comparison, some interesting information can be summarized. The optical fiber probe microcantilever sensor can be fabricated by a variety of methods, among which FIB milling technology has excellent machining accuracy but is time-consuming and has a high cost. The ps-laser ablation has a high processing efficiency while the processed surface quality is not enough. The cooperation of FIB milling and ps-laser ablation is a feasible manufacturing method for microcantilever-based fiber sensors. In addition, wire cutting is effective for the preliminary machining of millimeter-scale cantilever beams. The fs-laser micromachining and the fs-laser-induced TPP have obvious advantages in micro-nano processing. However, the price of an fs-laser is still relatively high at present. One possible solution is to develop suitable batch manufacturing processes. 

## 7. Conclusions and Outlook

With the continuous expansion of the optical communication market and the progress of micro/nano processing technology, optical fiber sensing technology is developing rapidly and is widely used in various fields. Due to the advantages of high sensitivity, small size, versatility, and resistance to harsh environments, cantilever-based optical fiber sensors have gradually become a research hotspot in the field of fiber devices. The cantilever-structured fiber optic interference sensor can fully expand the structure system and application range of fiber optic devices to meet the urgent demand for fiber optic devices with more functions and higher performance. This paper reviews different types of cantilever-based optical fiber interferometers including fiber-top cantilevers, ferrule-top cantilevers, and other cantilevers. The operation principle of cantilevers was described. The structural characteristics, fabrication methods, materials, and sensing applications of each cantilever-based interferometer sensor are introduced. However, it is reasonable to believe that the cantilever-structured fiber optic interference sensor has yet to reach its full potential, especially in commercial application. One important reason is that optical fiber microcantilever sensors are too fragile and are damaged by accidental collisions. Moreover, since the microcantilever probe is an open cavity, dust in the air will interfere with the light beam and affect the performance of the sensor or even make it completely ineffective. Therefore, the appropriate protection devices and packaging processes need to be further studied. 

At present, an obvious development trend for optical fiber microcantilever is to replace the traditional MEMS-based cantilever in biomolecule detection [97,98,99]. The functional coating technique of MEMS-based cantilevers can also be used in optical fiber microcantilever sensors. The latter provides a monolithic structure and simple interrogation approach, which gives it great application prospects in harsh environments and remote detection.

Another trend for optical fiber microcantilever sensors is gas sensing. Although some fiber microcantilever gas sensors based on photoacoustic spectroscopy have been reported, there are still many research directions to be explored, such as for volatile organic compounds, toxic gases, ammonia, ethanol, methanol, acetone, etc. [100,101,102,103]. Furthermore, optical fiber microcantilever gas sensors combined with photothermal cantilever deflection spectroscopy or chemical adsorption-based gas sensing technology could be exploited [104,105]. 

It is worth noting that optical fiber microcantilever sensors can be further developed into dual-parameter or multi-parameter sensing devices. Dual-parameter fiber microcantilever sensors have been demonstrated in the literature [46,83]. The design of a hybrid fiber sensor head is a crucial process. Multi-parameter fiber sensors become an attractive choice for reducing the total cost of sensing systems in complex practical applications. New functional materials, more efficient processing methods, and multi-parameter measurements are the future development directions of the cantilever structure fiber optic interference sensor.

## Figures and Tables

**Table 1:** Comparison of the optical fiber probe microcantilever sensor.

Sensor Structure	Fabrication Method	Microcantilever Dimension (μm)	Sensing Application	Microcantilever Material	Ref.
Fiber-top	FIB milling	112 × 14 × 3.7	AFM, hydrogen	silica	[41,50]
	chemical etching	100 × 16 × 20	displacement	silica	[54]
	ps-laser ablation	110 × 20 × 10	displacement	silica	[55]
	ps-laser + FIB	110 × 18 × 2	temperature, PH	silica	[43,56]
	photolithography	11 × 7 × 0.35	mass	gold	[57]
	TPP	20 × 20 × 3	hydrogen	polymer	[59]
Glass ferrule-top	ps-laser ablation	1600 × 200 × 30	air flows velocity	Borosilicate glass	[66]
	wire cut + ps-laser	2800 × 220 × 35	pressure, humidity	Borosilicate glass	[74]
Ceramic ferrule-top	ns-laser ablation	1400 × 300 × 25	food pathogen detection	Polyimide	[77]
	laser cutting	1000 × 500 × 5	microphone	Stainless steel	[78]
	MEMS	530 × 200 × 3	microphone	silica	[87]
Fiber-side	ps-laser + FIB	1000 × 35 × 6	Accelerometer	silica	[91]
SMF-GT- microcantilever	fs-laser micromachining	75 × 50 × 8	hydrophone	silica	[93]

## Data Availability

No new data were created or analyzed in this study. Data sharing is not applicable to this article.
